# Supplementary material for: “It is what we have been told to do”: Masculinities and femininities crossing with sexual orientation and feminist activism in Spain
Source: PLoS One. 2023 May 10;18(5):e0285544. doi: 10.1371/journal.pone.0285544 (PMC10171591; doi:10.1371/journal.pone.0285544)
Supplement: Attachment — Submitted filename: Response to Reviewers.docx [file pone.0285544.s001.docx]

**PONE-D-22-30849**

**“It is what we have been told to do”: Masculinities and femininities crossing with sexual orientation and feminist activism in Spain**

Dear Academic Editor Dr. Mary Diane Clark,

We thank you for the opportunity to submit the manuscript revised. We also would like to thank you and the reviewers for the valuable comments. Below we provide detailed responses on how we addressed each point raised during the review process. We have submitted a file labeled 'Revised Manuscript with Track Changes' with changes marked using red colored text, and a file labeled 'Manuscript' without tracked changes. We hope that these revisions will serve to meet PLOS ONE’s criteria.

Looking forward to hearing from you soon.

Kind regards,

Ariadna Cerdán-Torregrosa

**Response to Academic Editor comments:**

Journal Requirements:

When submitting your revision, we need you to address these additional requirements.

1. Please ensure that your manuscript meets PLOS ONE's style requirements, including those for file naming. The PLOS ONE style templates can be found at

<https://journals.plos.org/plosone/s/file?id=wjVg/PLOSOne_formatting_sample_main_body.pdf> and

<https://journals.plos.org/plosone/s/file?id=ba62/PLOSOne_formatting_sample_title_authors_affiliations.pdf>.

We have reviewed the documents listed and the other information available on the journal's website to ensure that our manuscript meets PLOS ONE's style requirements. A few changes have been made accordingly. Firstly, we have added full stops to the titles of Fig 1-3 and Table 1. Secondly, the naming of the figure files have been changed to "Fig 1.tif" and "Fig 2.tif" and we have made sure that our files comply with PLOS ONE's format.

2. Thank you for stating in your Funding Statement:

“This work was supported by GENDER NET Plus Co-Fund (Reference 2018-00968) and the Ministry of Science and Innovation of Spain (Reference PCI2019-103580) as part of the PositivMasc project from which CVC is the principal investigator. This study has also been conducted within the predoctoral grant received from the Ministry of Universities of Spain (Reference FPU19/00905) from which ACT is recipient.”

Please provide an amended statement that declares *all* the funding or sources of support (whether external or internal to your organization) received during this study, as detailed online in our guide for authors at <http://journals.plos.org/plosone/s/submit-now>.  Please also include the statement “There was no additional external funding received for this study.” in your updated Funding Statement.

Please include your amended Funding Statement within your cover letter. We will change the online submission form on your behalf.

We have amended the Funding Statement following your recommendations. The Funding Statement now is as follows:

“This work was supported by GENDER NET Plus Co-Fund (Reference 2018-00968) and the Ministry of Science and Innovation of Spain (Reference PCI2019-103580) as part of the PositivMasc project from which Carmen Vives-Cases is the principal investigator. This study was also conducted within the predoctoral grant received from the Ministry of Universities of Spain (Reference FPU19/00905) from which Ariadna Cerdán-Torregrosa is recipient. The publication costs were supported by AICO, Generalitat Valenciana (2022-2024) (Reference CIAICO/2021/019). The funders had no role in study design, data collection and analysis, decision to publish, or preparation of the manuscript. There was no additional external funding received for this study.”

3. Please review your reference list to ensure that it is complete and correct. If you have cited papers that have been retracted, please include the rationale for doing so in the manuscript text, or remove these references and replace them with relevant current references. Any changes to the reference list should be mentioned in the rebuttal letter that accompanies your revised manuscript. If you need to cite a retracted article, indicate the article’s retracted status in the References list and also include a citation and full reference for the retraction notice.

We have checked our cites and reference list to ensure that it is complete and correct and have detected the following error which has now been fixed: on line 585 the citation "[36]" has been changed to "[37]". We have not cited papers that have been retracted.

Additional Editor Comments (if provided):

thank you for. your manuscript. The reviewers made no suggestions but I have a few minor changes I would like to see. The topic is interesting and the work can be transformative.

We thank you for your enriching review of our manuscript and for the important comments you have made. Below we provide detailed answers for each of these comments, explaining how we have made changes in the manuscript based on them.

Line 413 Can you have a better conclusion to this section? It needs to have a summary rather then ending with a quote

Thank you for your suggestion. We have added the following paragraph between lines 414 and 416:

“In this sense, men are presented as free of responsibility and agency in contributing to the eradication of gender inequalities. This came with a pessimist perspective that gender inequalities would be impossible to eradicate.”

Line 420 can you delete the … after harassment and start the ( ) with i.e.,

Yes, we have modified the sentence in the light of your proposal. Please, see line 423:

“(i.e., rape, sexual abuse, psychological abuse, harassment)”

Also same on line 425

And the etc at the end of line 425

We have changed the sentence, please see lines 427-428:

“(i.e., clothing, waxing), profession choice, and enjoying certain leisure activities.”

Line 427 same three periods please add. Alternative punctuation

Agree. We have added alternative punctuation, please see line 430:

“who is blamed, the one who is ignored, the one who is made invisible, subject to the”

Line 426 ---gender binary. ---- should this be binary gender?

We have changed the term to "gender binarism" because participants refer to the social and cultural construct based on the belief that society has only two genders: feminine and masculine, as set out in the results. We hope that this change will make it clearer. Please, see line 429:

“of gender binarism, they explained that it corresponds to the worst side of things: the one”

Line 496---again you need a concluding paragraph

Agree. We have written a concluding paragraph, please see lines 498-501:

“Participants speak of a two-fold change. Firstly, and internal change regarding the way of understanding femininity from cooperation and diversity. Secondly, external change by accompanying other generations in this process of transformation towards equality.”

Line 562---again need a concluding paragraph

Yes. We have written a concluding paragraph accordingly, please see lines 568-572:

“However, people with this discourse recognize that abolishing gender requires an invaluable period of time due to the innumerable changes and transformations that must be carried out in the different spheres of society such as the educational system, the media or the legislative system. Some participants even go so far as to consider that achieving a genderless society is a utopia.”

**Response to Reviewer 1 comments:**

Reviewer #1: I enjoy reading this manuscript and it helped me to see why bisexuality is a vanishing point, because it does not fit the heteronormative dichotomy. However, I would recommend you to change this label LGB to LGBT as some transgender do internalize heteronormative tendencies.

Thank you very much for the review and your positive feedback. In accordance with your recommendation, we have modified the sentence in line 657:

“with LGBT activism in future studies, as well as how they may be in a unique position in”

However, we have kept the label "LGB" in line 690 because on that occasion we only referred to our sample, in which no transgender people participated.

**Response to Reviewer 2 comments:**

Reviewer #2: The submission "It is what we have been told to do": Masculinities and femininities crossing with sexual orientation and feminist activism in Spain is technically sound in its qualitative context using semi-structured interviews and discussion groups to identify a type of discourse that emerges in masculinities and femininities relative to sexual orientation and feminist activism in Spain. The data uncovered from the discussion groups supports the identification of a socially dominant gender discourse with the masculine approach being perceived as dominant over femininity. There also is a prevalence of reproducing that socially dominant gender discourse within non-activist heterosexuals, and the data are consistent with the understanding that there are others who try to deconstruct that gender discourse who are activists across all sexual orientations.

In terms of statistical analysis - there is no quantitative analysis presented - which is appropriate given the qualitative context of the study. Qualitative analysis was used via Atlas.ti (qualitative analysis platform) - where the authors analyzed the "narrative configurations and discursive positions" of the participants. The first author led the analytic process with the remaining authors, engaging in a continuous discussion process where guidance and feedback was provided to ensure consistency within the rigor and validity of the sociological analysis.

The results from the analysis are consistent with the identification of a socially dominant gender discourse and are explained clearly by group (seven data groups in all) using paraphrases from interviews as forms of evidence to support the identification of that gender discourse.

Finally, future direction is provided in identifying the need to continue working on a "gender-transformative approach through interventions from an early age as well as deconstructing the biological beliefs about gender roles to amplify and promote gender equality/social justice."

We really appreciate the time you have dedicated to our manuscript. Thank you very much for the review and your valuable feedback.
